# The impact of hypercortisolism beyond metabolic syndrome on left ventricular performance: a myocardial work analysis

**DOI:** 10.1186/s12933-025-02680-1

**Published:** 2025-03-21

**Authors:** Floran Sahiti, Mario Detomas, Vladimir Cejka, Kristina Hoffmann, Götz Gelbrich, Stefan Frantz, Mathias Kroiss, Peter U. Heuschmann, Stefanie Hahner, Martin Fassnacht, Timo Deutschbein, Stefan Störk, Caroline Morbach

**Affiliations:** 1 Department of Clinical Research and Epidemiology, Comprehensive Heart Failure Center, University Hospital Würzburg, Würzburg, Germany; 2 Division of Cardiology, Department of Internal Medicine I, University Hospital Würzburg, Würzburg, Germany; 3 Division of Endocrinology and Diabetes, Department of Internal Medicine I, University Hospital Würzburg, Würzburg, Germany; 4 Institute for Clinical Epidemiology and Biometry, University of Würzburg, Würzburg, Germany; 5 Institute of Medical Data Science, University Hospital Wurzburg, Würzburg, Germany; 6 Department of Internal Medicine IV, University Hospital Munich, Ludwig Maximilians-Universität München, Munich, Germany; 7 Medicover Oldenburg MVZ, Oldenburg, Germany

**Keywords:** Myocardial work, Cushing's syndrome, Metabolic syndrome, Cortisol, Left ventricular performance

## Abstract

**Background and aims:**

Endogenous Cushing's syndrome (CS) is characterized by an unfavorable cardiovascular (CV) and metabolic risk profile, but the potential adverse effects of hypercortisolism on myocardial function are not well known. Myocardial Work analysis is a new echocardiographic method that utilizes left ventricular pressure-strain loops to quantify cardiac performance independent of afterload.

**Methods and results:**

In a cross-sectional analysis, we compared four groups: patients with overt endogenous CS (n = 31, mean age 47 ± 12 years, 71% women), patients with endogenous CS in long-term remission after medical cure (CS-LTR; n = 49, 53 ± 12 years, 78% women), healthy subjects (n = 439; 49 ± 11 years, 57% women), and individuals with metabolic syndrome (n = 305, 59 ± 10 years, 37% women). Both CS patient groups exhibited a CV risk pattern and metabolic profile worse than healthy subjects but better than individuals with metabolic syndrome. Analyses adjusted for sex and age revealed higher Wasted Work both in overt CS (median; quartiles: 105 mmHg%; 74, 147) and CS-LTR (97 mmHg%; 69, 158), respectively, when compared to healthy individuals (75 mmHg%; 54, 109, *p* < 0.01) or individuals with metabolic syndrome (95 mmHg%, 65, 136, *p* < 0.05), resulting in compromised Work Efficiency (*p* < 0.05).

**Conclusion:**

Left ventricular performance is compromised in overt CS beyond alterations found in individuals with metabolic syndrome sharing equal CV risk factors and remains so despite biochemical remission during the LTR period. Myocardial Work analysis is suited to detect the subtle yet clinically relevant differences between different phenotypes of myocardial involvement.

**Supplementary Information:**

The online version contains supplementary material available at 10.1186/s12933-025-02680-1.

## Introduction

Endogenous Cushing's syndrome (CS) is a rare but aggressive endocrine disorder characterized by glucocorticoid excess leading to an unfavorably altered metabolism [1–3], and the manifestation of conditions constituting the metabolic syndrome (MS), i.e., arterial hypertension, diabetes mellitus, dyslipidemia, and obesity [4, 5]. Numerous reports associated CS with atherosclerosis, LV remodeling, myocardial fibrosis, and LV dysfunction, and reported a subsequently increased risk of cardiovascular (CV) morbidity and mortality [6–13], most likely associated with the presence of CV risk factors. Nevertheless, recent findings point towards an adverse effect of hypercortisolism beyond the impact of CV risk factors[14, 15]. Previous studies have shown myocardial structural abnormalities [10], such as an increase in relative wall thickness and LV hypertrophy, which are precursors of heart failure (HF) [12, 16, 17]. However, overt HF is infrequent in CS and is mainly associated with the phenotypes of a preserved LV ejection fraction (LVEF) or a subclinical LV dysfunction [4, 18, 19].

Treatment of endogenous CS targets to eliminate glucocorticoid excess and frequently includes a combination of surgery, radiation, and pharmacotherapy. Long-term treatment aims to provide a balanced endocrine profile and may involve the substitution of various hormones including corticosteroids. Of note, studies have consistently shown that CV mortality remains elevated even in patients with endogenous CS who are regarded in biochemical long-term remission (LTR) under the best medical care [4, 20].

In patients with CS, echocardiography is recommended both at the initial presentation and during follow-up [21]. In these patients, conventional echocardiographic parameters typically are still within normal ranges, but they deviate from the general population mean which is consistent with an elevated CV risk [14]. However, earlier work suggested that the compromise of LV performance caused by hypercortisolism evades the standard hemodynamic assessment [22]. Therefore, new diagnostic tools are needed that are more sensitive to the subtle but clinically relevant changes in LV performance that may be caused by an unfavorable endocrine and metabolic profile.

Myocardial Work (MyW) analysis is a novel echocardiographic method based on the measurement of pressure-strain loops [23–25]. By accounting for both the effective work contributing to contraction and early relaxation, as well as the inefficiency of work expended during these phases, MyW offers a comprehensive evaluation of LV performance [24]. It complements conventional parameters such as LVEF and measures of diastolic dysfunction, extending insights into both global and segmental cardiac function. Taking blood pressure into consideration, MyW is considered less load-dependent than LVEF and global longitudinal strain (GLS) [25–27].

The aim of this study was to employ MyW to identify the potentially subtle differences in LV performance in patients with overt CS and CS in LTR and to compare these findings with those obtained in samples derived from the general population, namely healthy subjects (HS) and individuals with MS, the latter sharing equal CV risk factors with CS patients.

## Methods

### Studied samples

#### Endogenous Cortisol Excess Study (CV-CORT-EX)

The Endogenous Cortisol Excess (CV-CORT-EX) [28] cohort study was designed to characterize the effects of endogenous hypercortisolism on cardiac morphology and function [28]. The study was jointly conducted by the Division of Endocrinology, Division of Cardiology, and the Comprehensive Heart Failure Centre (CHFC) of the University Hospital Würzburg, and comprised a cross-sectional and a longitudinal arm [28]. Details of the study design and methodology have been published [28]. Patients provided written informed consent at the time of study enrollment. The study complied with the Helsinki Declaration and was approved by the Ethics Committee of the University Hospital Würzburg (NCT number: NCT03880513).

Based on the current guidelines [1, 29, 30], participants of the CV-CORT-EX study included in this analysis were categorized as 1) overt CS or 2) cured CS (i.e., formerly overt CS currently in biochemical remission (CS in LTR)) [1, 29, 30]. All patients underwent a comprehensive standardized diagnostic work-up including medical history (focused on CV risk factors and endocrine disorders), clinical evaluation, biochemical characterization, and imaging studies (including detailed transthoracic echocardiography) [28]. The presence and remission of CS was confirmed by serum cortisol levels measured during the 1-mg dexamethasone suppression test (DST), salivary cortisol day profiles, and 24-h urinary-free cortisol [1, 28].

#### Characteristics and Course of Heart Failure STAges A/B and Determinants of Progression (STAAB)

The STAAB study recruited and characterized a representative sample of the population of Würzburg, Germany, aged 30–79 years, stratified for age and sex, free of symptomatic heart failure [16, 31]. Participants underwent a comprehensive workup including standardized physical examination, physician interview, laboratory testing, and transthoracic echocardiography examination according to standard operating procedures. The STAAB study was planned and carried out by a multidisciplinary team of researchers from the University of Würzburg.

From STAAB, two subgroups were defined. Subgroup 1 comprised HS defined by the following profile: no CV risk factors, presence of sinus rhythm, LVEF above 50%, and no significant valvular disease. Subgroup 2 comprised subjects exhibiting MS defined by the presence of at least three of the following conditions: (a) waist circumference ≥ 102 cm in men or ≥ 88 cm in women, (b) triglycerides ≥ 1.7 mmol/L and/or on medication for high triglycerides, (c) high-density lipoprotein < 40 mg/dL in men or < 50 mg/dL in women, and/or on lipid-modifying drug, d) systolic blood pressure ≥ 130 mmHg or diastolic blood pressure ≥ 85 mmHg, e) fasting blood glucose ≥ 6.1 mmol/L.

### Echocardiography and assessment of Myocardial work

In both CV-CORT-EX and STAAB, transthoracic echocardiography (using Vivid S6, Vivid E9, Vivid E95, GE Healthcare, Horton, Norway) was conducted at the Comprehensive Heart Failure Center (CHFC) Würzburg based on standard operating procedures [32] and regular quality checks [33]. Two-dimensional images were recorded with a frame rate of 50–80 s^−1^ and stored digitally.

The step-by-step process on how to conduct MyW analysis that has been published by our group [16, 34] was also utilized for the current study. Based on blood pressure measurements taken immediately prior to the echocardiography examination and the stored echocardiography images, the following MyW indices were derived (EchoPAC®, Version 202, GE):

1. Global constructive work [GCW (mmHg%)], which, i.e., the sum of positive work (shortening) performed during systole and adding the amount of negative work (lengthening) during isovolumic relaxation time;
2. Global wasted work [GWW (mmHg%)], i.e., the sum of negative work (lengthening) performed during systole plus positive work (shortening), performed against a closed aortic valve during isovolumic relaxation time;
3. Global work index [GWI (mmHg%)], which is a surrogate of the total work performed from mitral valve closure to mitral valve opening.;
4. Global work efficiency [GWE (%)], which was computed using the formula: $GWE=\frac{\text{GCW}}{(\text{GCW}+\text{GWW})}$

All indices were calculated as the mean of the respective segmental values applying an 18-segment model. Subjects in whom > 1 LV segment was unsuitable for analysis due to poor tracking or suboptimal image quality were excluded from further analysis. Previous data from our group had revealed favorable inter- and intraobserver variability regarding MyW parameters [31, 35].

### Data analysis

Statistical analysis was performed using SPSS (Version 26, SPSS Inc., Chicago, USA). Descriptives of continuous variables are provided as means (standard deviation) or median (quartiles), as appropriate. Categorical variables are presented as frequencies (percent). The variables were assessed for normality using the Shapiro–Wilk test. Differences in normally and non-normally distributed variables were evaluated with Student´s t-test, and the Mann–Whitney U-test, respectively. The Chi-square test was used to compare categorical variables. Analysis of covariance (ANCOVA) adjusted for age, sex, body mass index (BMI), and systolic blood pressure was used to assess differences in MyW between the entities. *P* values < 0.05 were considered statistically significant.

## Results

For the CV-CORT-EX study, 114 patients with current or previous endogenous glucocorticoid excess were recruited and comprehensively characterized [14]. For the cross-sectional analysis of the current study, we considered the 80 patients in whom MyW analysis could be conducted. Their mean age was 51 ± 12 years and 75% were women. Of these patients, 31 had overt CS (age 47 ± 12 years, 71% women), and 49 patients suffered from CS in LTR (age 53 ± 12 years, 77% women), with a median of 88 (quartiles 34, 182) months after cure. In the two patient cohorts with either overt CS or CS in LTR, pituitary tumors were the most common entity (39% and 57%), followed by tumors located in the adrenals (39% and 37%) or ectopic (22% and 6%; Table 1). The sources of ectopic tumors in overt CS were identified in five cases (lung carcinoid n = 3, pancreatic neuroendocrine tumor (n = 1), medullary thyroid cancer (n = 1). Additionally, in two patients no tumor could be detected. There were no significant differences in age, body mass index, systolic blood pressure, kidney function, NT-proBNP, or 24-h urinary free cortisol levels when comparing patients with overt ectopic CS to those with overt pituitary or adrenal CS. However, patients with overt ectopic CS were more frequently of male sex, had higher HbA1c, and lower potassium and LDL levels when compared to the other two subgroups. All except one patient with overt CS were investigated at the time of their initial tumor diagnosis. In detail, this patient with Cushing’s disease had a recurrence one year after surgery and was treated with pasireotide 0.6 mg twice daily at the time of evaluation. As normalization of 24-h urinary free cortisol was not achieved, the dose was subsequently increased to 0.9 mg twice daily. For comparison, we analyzed HS, n = 439, age 49 ± 11 years, 56% women, and individuals with MS, n = 295, age 60 ± 10 years, 37% women. Further details on the study cohort are given in Table 1.

**Table 1:** Characteristics of patients with Cushing’s syndrome (either overt or in Long-Term Remission) and healthy subjects without or with metabolic syndrome

	Overt CS, n = 31	CS in LTR, n = 49	STAAB HS, n = 439	STAAB MS, n = 295
Age, (years)	47 (12)	53 (12)	49 (11)^a^	60 (10)^C, c^
Women	22 (71)	38 (78)	248 (57)^b^	109 (37)^C, c^
BMI (kg/m^2^)	28 (6)	27 (6)	24 (3)^C,c^	31 (4)^C, c^
SBP (mmHg)	142 (24)^a^	131 (17)^A^	122 (11)^C, c^	139 (16)^A^
DBP (mmHg)	86 (18)	79 (10)	75 (8)^C, b^	82 (10)^a^
NT-proBNP (pg/ml)	65 (42, 98)	81 (50, 166)	42 (22, 82)^C, c^	57 (27, 102)^C, b^
LDL (mg/dL)	118 (46)	126 (30)	117 (30)	123 (36)
HbA1c (%)	6.0 (1.1)^b^	5.6 (0.6)^B^	5.3 (0.3)^C, b^	6.0 (0.9)^a^
Tumor source of endogenous Cushing´s syndrome				
Pituitary, n, (%)	12 (39)	28 (57)	–	–
Adrenal, n, (%)	12 (39)	18 (37)	–	–
Ectopic, n, (%)	7 (22)	3 (6)	–	–
Serum cortisol (after 1 mg Dexamethasone), (μg/dl)	14.3 (6.2, 21.2)	1.0 (1.0, 1.3)	–	–
24h urinary free cortisol (mcg/24h)	194 (53, 385)	41 (27, 78)	–	–
Glucocorticoid substitution n, (%)	0 (0)	35 (71	–	–
Comorbidities				
Hypertension n, (%)	23 (74)^b^	24 (49)^B^	–	243 (82)^c^
Diabetes mellitus n, (%)	12 (39)^b^	6 (12)^B^	–	92 (31)^b^
Hypothyreosis	12 (39)	23 (47)	–	–
Osteoporosis	9 (29)	9 (18)^B^		
Medications				
Beta blocker, n (%)	9 (29)	10 (20)	–	92 (31)
ACE or AT1 antagonist, n (%)	16 (52)	16 (33)^A^	–	138 (48)
Diuretics, (%)	7 (23)	6 (12)	–	40 (15)
Aldosterone antagonist, (%)	2 (7)	1 (2)	–	–
Echocardiography				
IVSd (mm)	9 (1)^a^	9 (1)^A^	8 (1)^C, b^	10 (1)^b^
LVEDd (mm)	47 (5)	45 (5)	47 (5)	50 (5)^b^
LVPWd (mm)	8 (1)^a^	8 (1)^A^	7 (1)^C, b^	9 (1)^a^
LVEDV (mL)	65 (17)	81 (18)	97 (25)^C, c^	106 (24)^C, c^
E/e’	9 (3)	9 (3)	7 (2)^C, c^	9 (3)
Left atrial volume index (mL/m^2^)	19 (15, 24)	20 (15, 27)	22 (18, 26)^B^	25 (20, 31)^B, b^
LVEF (%)	62 (4)	61 (6)	61 (4)	60 (5)
GLS (–%)	19.3 (2.5)	20.4 (2,4)	21.4 (2.4)^C,c^	19.1 (2.4)

Data are n (%) or mean (SD) or median (interquartile) respectively. *P*-values adjusted for age and sex

BMI, body mass index; CS, Cushing Syndrome; DBP, diastolic blood pressure; E/e’, ratio between early mitral inflow velocity and mitral annular early diastolic velocity; GLS, global longitudinal strain; HbA1c, glycosylated hemoglobin; HS, healthy subjects; IVSd, interventricular septum diameter; LDL, low-density lipoprotein; LTR, long-term remission; LVEDd, left ventricular end-diastolic diameter; LVEDV, left ventricular end-diastolic volume; LVEF, left ventricular ejection fraction; LVPWd, left ventricular poster wall diameter; MS, metabolic syndrome; NT-proBNP, N-terminal pro-natriuretic peptide; SBP, systolic blood pressure

Comparison versus overt CS: A *p* < 0.05, B *p* < 0.01, C *p* < 0.001; comparison versus CS in LTR: a *p* < 0.05, b *p* < 0.01, c *p* < 0.001

### Overt CS versus HS and MS

Patients with overt CS had a similar age and sex distribution as HS, but were significantly younger and more frequently women than MS (Table 2). Analysis of covariance adjusted for age and sex revealed that patients with overt CS exhibited higher BMI, higher systolic and diastolic blood pressure, higher levels of NT-proBNP, HbA1c, and larger LV volume when compared to HS. Furthermore, they exhibited lower BMI, smaller left atrial volumes, and slightly higher systolic blood pressure than HS and MS. In comparison to MS, overt CS exhibited lower BMI and slightly higher systolic blood pressure. There was no significant difference in LVEF between overt CS and HS or MS. In contrast, GLS was less favorable in patients with overt CS when compared to HS, whereas this was not the case when compared to MS. Furthermore, patients with overt CS presented with higher blood pressure values, elevated HbA1c, and a higher prevalence of diabetes mellitus compared to patients with CS in LTR. However, there was no difference in markers of LV systolic and diastolic function (Table 1).

**Table 2:** Myocardial work in Cushing’s syndrome (either overt CS or in CS in long-term remission) and healthy subjects without or with metabolic syndrome

	Overt CS n = 31	CS-LTR n = 49	STAAB Healthy subjects n = 439	STAAB Metabolic syndrome n = 293
GCW (mmHg%)	2581 (455)	2514 (378)	2440 (334)^B, a^	2483 (423)
GWW (mmHg%)	105 (74, 147)	97 (69, 158)	75 (54, 109)^B, c^	95 (65, 136)^A, b^
GWI (mmHg%)	2290 (387)	2265 (378)	2224 (310)^C, b^	2244 (400)
GWE (%)	95 (2)	95 (3)	96 (2)^B, c^	95 (2)^a^

Data are n (%), mean (SD), or median (quartiles), respectively

*p*-values are obtained from analysis of covariance (ANCOVA) adjusted for age, sex, body mass index, and systolic blood pressure

Comparison versus overt CS: A *p* < 0.05, B *p* < 0.01, C *p* < 0.001

Comparison versus CS in LTR: a *p* < 0.05, b *p* < 0.01 c, *p* < 0.001

CS, Cushing`s syndrome; GCW, global constructive work; GWE, global work efficiency; GWI, global work index; GWW, global wasted work; LTR, long-term remission

### CS in LTR versus HS and MS

Patients with CS in LTR were older and more frequently women compared to HS. They exhibited a higher BMI, higher blood pressure, higher NT-proBNP, larger LV wall thickness, lower LV volume, and less favorable GLS compared to HS. Besides, they were younger, more frequently women, exhibited smaller left atrial volumes although mean values were still within the normal range, lower values of blood pressure, higher values of NT-proBNP, more favorable LV dimensions, and no difference in systolic and diastolic function when compared to MS (Table 1).

### Overt CS versus CS in LTR

We found no significant differences between overt CS and CS in LTR regarding age, sex, BMI, E/e`, LVEF, or GLS. However, patients with overt CS had more frequent hypertension and diabetes mellitus.

### MyW analysis

**Fig. 1:**
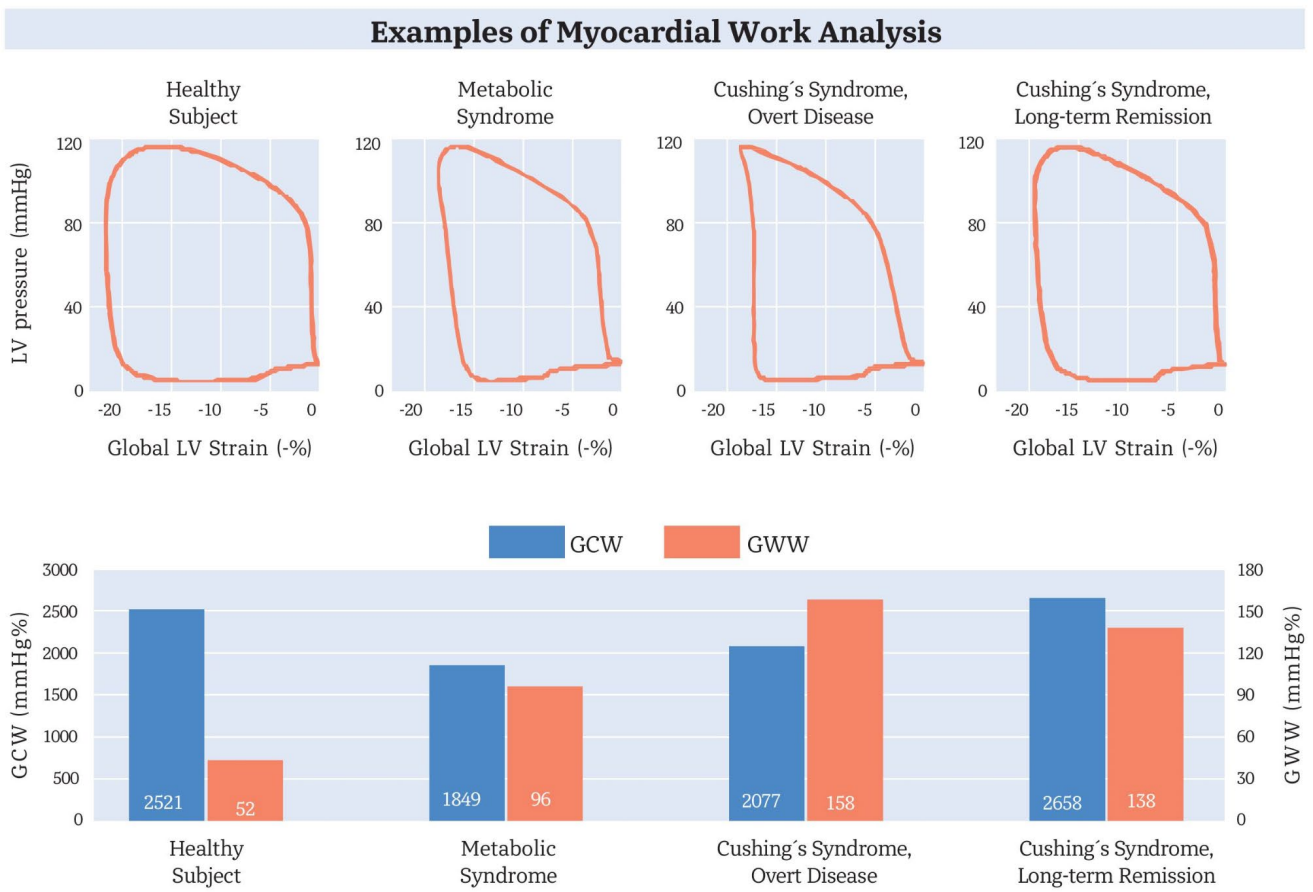
MyW analyses of exemplary study participants of the different entities included in our study. The upper part shows pressure-strain loops demonstrating the amount of work performed by the left ventricle. Please note that the blood pressure levels of all participants were similar, and there were only minimal differences regarding global LV strain (see Table 1). In the lower part, the blue area represents the constructive work of the LV, while the orange area indicates the wasted work (for respective definitions please refer to Methods). GCW, global constructive work; GWW, global wasted work; LV, left ventricular

After adjusting for possible covariates such as age, sex, body mass index, and blood pressure, CS patients with overt disease or in LTR revealed compromised LV performance as measured by increased GWW and decreased GWE (Table 2). Further, patients with overt CS exhibited more impaired MyW than individuals with MS. These differences were less pronounced when compared to patients with CS in LTR (Table 2). An illustrative example of the MyW pressure-strain loop in the entities included in our study are shown in Fig. 1.

### Correlation of MyW indices with CV risk factors and 24-urinary free cortisol (UFC)

Only blood pressure showed a significant association with MyW parameters, while other CV risk factors and 24-h UFC had no significant associations with MyW indices (Table 3).

**Table 3:** Correlation of MyW parameters with CV risk factors and 24h urinary free cortisol in patients with Cushing’s syndrome (overt or in long-term remission)

Variable	GCW		GWW		GWI		GWE	
	Spearman	P	Spearman	P	Spearman	P	Spearman	P
Body mass index (kg/m^2^)	– 0.022	0.845	0.007	0.954	0.036	0.752	– 0.064	0.575
Systolic blood pressure (mmHg)	0.576	< 0.001	0.455	< 0.001	0.505	< 0.001	– 0.337	< 0.002
HbA1c (%)	0.043	0.709	0.127	0.264	0.069	0.547	– 0.132	0.248
24-h urinary free cortisol (μg/24 h)	0.030	0.796	0.212	0.062	– 0.030	0.793	– 0.183	0.108
Serum cortisol (after 1 mg dexamethasone) (μg/dl)	0.052	0.660	0.133	0.257	0.001	0.994	– 0.119	0.315

GCW, global constructive work; GWE, global work efficiency; GWI, global work index; GWW, global wasted work; LTR, long-term remission

## Discussion

In the present cross-sectional work, we investigated LV performance using MyW analysis in a well-characterized cohort of patients with current and previous endogenous glucocorticoid excess and compared to HS and individuals with MS, derived from a representative sample of the general population of the city of Würzburg. Here, we report the following findings: Firstly, in contrast to LVEF as an established parameter of cardiac function, even after adjusting for potential confounders (age, sex, BMI, and blood pressure), MyW analysis revealed functional alterations in both CS patient groups when compared to HS and individuals with MS. Patients with endogenous CS exhibited higher wasted work, even during the LTR period. Secondly, MyW was more impaired in both CS patient groups than in individuals with MS, suggesting an adverse effect of glucocorticoid excess on LV performance beyond the effects related to the presence of CV risk factors. Thirdly, our findings suggest CS patients experience prolonged cardio-functional impairment despite biochemical cure.

### Conventional and novel echocardiographic parameters in Cushing's syndrome

Despite its inherent constraints, LVEF remains the most frequently utilized parameter for evaluating LV function. The majority of studies on patients with CS exhibited a preserved LVEF [15, 22]. In these studies, cardiac affections were predominantly manifested in LV morphology and geometry [15], indicating only mild and therefore clinically inconsequential alterations of LV performance [8, 15]. Yet, the long-term relevance of such more subtle alterations may have been underestimated in patients with CS. Previous smaller studies [10, 18, 22, 36] investigated GLS in participants with CS, patients with arterial hypertension, and healthy controls and showed that GLS was only significantly reduced in CS patients. The normalization of cortisol levels was associated with an improvement in LV longitudinal function [18], and this observation was independent of changes in blood pressure [4]. Our findings regarding GLS are not only consistent with prior data [36] but also show that the alterations in CS patients were regardless of age, sex, blood pressure, and BMI. However, in comparison to individuals with MS, we found no differences regarding GLS.

We applied MyW analysis as a novel echocardiography method, thereby revealing functional alterations in patients with current and previous glucocorticoid excess when compared to HS. These alterations involved a larger amount of work performed in general, (including wasted work), but lower work efficiency. These alterations persisted even during LTR (but less pronounced when compared to the patients with overt CS) and were regardless of blood pressure and BMI, indicating impaired LV performance even after long-term biochemical cure of CS and most likely contributing to an increased mortality rate in affected patients [10, 37, 38]. Further, in different populations, MyW has been found to be associated with exercise capacity [39, 40]. Increased wasted work and reduced myocardial work efficiency have been shown to be associated with impaired aerobic exercise capacity [41].

### LV performance beyond secondary effects of metabolic syndrome

Given the clinical overlap between MS on one hand and CS on the other, we aimed to determine, whether excess glucocorticoid excess impacts LV performance beyond the secondary effects of MS. In our population, 15% of individuals presented with MS, which is consistent with findings from other studies [42, 43]. Previously, we demonstrated that each CV risk factor affects LV performance in distinct ways [31], also leading to LV remodeling [16, 17]. LV remodeling is also a possible consequence of glucocorticoid excess [3].

In line with previous studies [3, 28], we found prevalences for hypertension of 74% (in overt CS), 49% (in CS in long-term remission), and 82% (in MS). In one of our earlier works, we demonstrated that hypertension was the primary risk factor significantly affecting LV performance [31]. Some studies reported that cortisol levels can predict changes in LV morphology independently of blood pressure [44, 45]. Of note, it is known that cortisol itself exerts diverse effects on the cardiovascular system [2, 8]. The mechanism behind the LV changes remains however not completely understood since pathophysiological studies about this topic are still lacking. Treatment of cortisol excess and restoration of normal cortisol levels showed an improvement in comorbidities [3, 17, 46, 47] resulting in normalized cardiac function [4, 18], and decreased cardiovascular mortality rates [20]. However, despite optimal treatment of cortisol excess and comorbidities, certain changes persisted in CS patients in LTR [4]. Further, despite biochemical remission for up to ten years, mortality remained high [11, 48]. These changes remain consistent, regardless of the blood pressure levels observed during follow-up, indicating that hypertension only partly accounts for the alterations in LV morphology and performance in CS patients [17–19, 45].

Further, another study found no direct correlation between LV mass or LV hypertrophy with blood pressure levels in patients with CS [8]. The interaction of hypertension and cortisol increases the risk of LV remodeling i.e., by activating and enhancing components of the renin-angiotensin system and receptors in myocytes [10], thus CS patients with hypertension had a 2-times higher risk of LV remodeling compared to those without hypertension [15]. LV remodeling in CS patients might also arise due to indirect mechanisms, including impaired coronary microvascular function and increased vascular stiffness [49], often leading to myocardial fibrosis [10, 22]. In previous work, which included individuals from the general population, we demonstrated that CV risk factors adversely affect myocardial work, independent from systolic blood pressure, individually, and in a sex-specific manner [31]. There, hypertension emerged as the most powerful risk factor associated with altered LV performance [16, 31]. In line, we found a strong association of hypertension with impaired MyW. However, in this study, other factors like diabetes mellitus and cortisol levels showed no significant association with MyW indices, most likely due to the small sample size. A study showed that patients with overt CS showed increased myocardial fibrosis [10], compared to matched individuals with LV hypertrophy and hypertension and healthy controls [10]. Myocardial fibrosis is considered to be the most important ultrastructural abnormality in CS cardiomyopathy [3, 10]. Potential mechanisms of action have been described previously [50, 51]. Cortisol exerts its effects through glucocorticoid receptors and mineralocorticoid receptors [50]. The activity of these receptors is influenced by the presence of 11β-hydroxysteroid dehydrogenase type 1 (11β-HSD1) and type 2 (11β-HSD2) enzymes, which vary across different tissues [50]. Cardiomyocytes exhibit very low expression of 11β-HSD2 [50, 52, 53]. In healthy tissues, they help to regulate normal cortisol levels [50, 51]. However, in damaged tissues where 11β-HSD2 is absent, MRs may be occupied by cortisol, which also acts as an MR agonist, particularly under conditions of elevated cortisol levels [51]. This cascade of events promotes myocardial fibrosis, leading to cardiac dysfunction [50]. During follow-up, a significant reduction of myocardial fibrosis and improvement of LV function after normalization of cortisol was observed [10]. In line, another study showed that upon remission of hypercortisolism, most LV abnormalities were markedly reduced [15, 19]. MyW analysis was already used to assess myocardial fibrosis, especially in patients with hypertrophic cardiomyopathy (HCM), showing that GWI, GCW, and GWE, remained significant predictors of LV myocardial fibrosis [54]. Using echocardiography, we cannot determine the presence of fibrosis, however, our data show a more impaired LV performance during overt CS.

In our study, the metabolic profile of CS patients in LTR was better than in patients with overt CS but worse than in HS. Consistently, CS patients in LTR showed worse LV performance than HS, with a higher proportion of wasted myocardial work. MyW in both CS patient groups was more impaired also when compared to participants with MS, suggesting an adverse effect of cortisol excess on LV performance beyond the effects of secondary to metabolic alterations (despite having a better metabolic situation and younger age). Our data highlight the significant and persistent adverse effects of cortisol excess on LV performance, detectable through MyW analysis. This underscores the need for careful cardiovascular monitoring and management in patients with current and former cortisol excess, even after achieving biochemical remission.

Patients with ectopic CS represent a small subset of endogenous glucocorticoid excess. In line with previous studies [55, 56], our study found that male sex was more frequent in ectopic CS, whereas female sex was more frequent in the other CS subtypes. However, apart from higher levels of HbA1c and lower levels of potassium and LDL, no significant differences in clinical characteristics were observed. Sex-related differences in CS have been previously investigated [55, 57, 58]; however, the underlying pathophysiological mechanisms remain unclear. A possible explanation might be that men have a higher incidence of malignant diseases in general and of carcinoid tumors in particular [55]. However, our study was not designed to provide distinct pathophysiological insights, and further research is needed to investigate sex-specific differences in patients with CS. Nevertheless, using MyW as a less load-dependent method to assess myocardial performance may prove to be a promising tool for early detection and monitoring of cardiac alterations in patients with CS. Therefore, follow-up studies are needed to further explore its potential.

### Limitations

We acknowledge the small sample size as a major limitation. This is particularly true when subdividing the whole cohort into smaller subgroups (e.g., overt CS and CS in LTR). Nevertheless, given the rarity of the disease, our study population, recruited within CV-CORT-EX, can be regarded as relatively large compared to other studies published on this topic to date. Owing to the cross-sectional design of the study, it was not possible to infer causality for the observed associations. Further, longitudinal studies are needed to allow for the tracking of changes in myocardial function in CS patients over time. Additionally, our study was not designed to explore the potential underlying pathophysiological mechanisms; the roles of inflammation and cortisol certainly warrant deeper investigation. The absence of functional outcome measures, such as exercise capacity or quality of life assessments, restricts a more comprehensive understanding of the clinical relevance of our findings. Furthermore, we acknowledge that the long median duration after remission in the CS-LTR group might have introduced selection bias, possibly limiting the generalizability of the results. Lastly, the generalizability of the findings may be constrained due to the specific regional characteristics of the study population, highlighting the importance of examining the effects of CS in different ethnic or socioeconomic groups in the future. Of note, one of the notable strengths of this study is the rigorous and consistent echocardiographic workup conducted in both studies, employing standardized operating procedures with the involvement of the same investigators, machines, and readers. This standardized approach enhances the reliability and validity of our findings.

## Conclusions

Myocardial work analysis reveals significant alterations in LV performance in patients with current and previous cortisol excess, highlighting increased wasted work not only in the acute phase but also in the long-term. Patients with overt CS exhibited more impaired MyW than individuals with MS, indicating that glucocorticoid excess might have a detrimental impact on myocardial performance beyond metabolic alterations. Further studies are needed to validate our findings and identify targets for enhancing metabolic situation and cardiac function in patients with biochemically cured CS.

## Electronic supplementary material

Below is the link to the electronic supplementary material.

Supplementary Material 1

## Data Availability

The data that support the findings of this study are not openly available due to reasons of sensitivity and are available from the corresponding author upon reasonable request.

## Abbreviations

CS: Cushing's syndrome
CV-CORT-EX: Endogenous Cortisol Excess Study
GCW: Global constructive work
GLS: Global longitudinal strain
GWE: Global work efficiency
GWI: Global work index
GWW: Global wasted work
HFpEF: Heart failure with preserved ejection fraction
HFrEF: Heart failure with reduced ejection fraction
LVEF: Left ventricular ejection fraction
LTR: Long-term remission
MACS: Mild autonomous cortisol excretion
MS: Metabolic syndrome
MyW: Myocardial work
STAAB: Characteristics and Course of Heart Failure STAges A/B and Determinants of Progression
