# Supplementary material for: The impact of hypercortisolism beyond metabolic syndrome on left ventricular performance: a myocardial work analysis
Source: Cardiovasc Diabetol. 2025 Mar 21;24:132. doi: 10.1186/s12933-025-02680-1 (PMC11929293; doi:10.1186/s12933-025-02680-1)
Supplement: Supplementary file 1 — Supplementary Material 1 [file 12933_2025_2680_MOESM1_ESM.docx]

**Supplementary Table 1.** Characteristics of patients with Cushing’s syndrome (either overt or in Long-Term Remission) and healthy subjects without or with metabolic syndrome.

|  | Overt CS, n=31 (1) | CS in LTR, n=49 (2) | STAAB HS, n=439 (3) | STAAB MS, n=295 (4) | 1 vs. 2 | 1 vs. 3 | 1 vs. 4 | 2 vs. 3 | 2 vs. 4 |
| --- | --- | --- | --- | --- | --- | --- | --- | --- | --- |
| Age, [years] | 47 (12) | 53 (12) | 49 (11) | 60 (10) | 0.071 | 0.529 | <0.001 | 0.018 | <0.001 |
| Women | 22 (71) | 38 (78) | 248 (57) | 109 (37) | 0.508 | 0.115 | <0.001 | 0.005 | <0.001 |
| BMI [kg/m2] | 28 (6) | 27 (6) | 24 (3) | 31 (4) | 0.746 | <0.001 | <0.001 | <0.001 | <0.001 |
| SBP [mmHg] | 142 (24) | 131 (17) | 122 (11) | 139 (16) | 0.018 | <0.001 | 0.041 | <0.001 | 0.077 |
| DBP [mmHg] | 86 (18) | 79 (10) | 75 (8) | 82 (10) | 0.058 | <0.001 | 0.500 | 0.006 | 0.024 |
| NT-proBNP [pg/ml] | 65 (42, 98) | 81 (50, 166) | 42 (22, 82) | 57 (27, 102) | 0.072 | <0.001 | <0.001 | <0.001 | 0.190 |
| LDL [mg/dL] | 118 (46) | 126 (30) | 117 (30) | 123 (36) | 0.373 | 0.600 | 0.101 | 0.512 | 0.766 |
| HbA1c [%] | 6.0 (1.1) | 5.6 (0.6) | 5.3 (0.3) | 6.0 (0.9) | 0.002 | <0.001 | 0.140 | 0.007 | 0.021 |
| *Tumor source of endogenous Cushing´s syndrome* |  |  |  |  |  |  |  |  |  |
| Pituitary, n, [%] | 12 (39) | 28 (57) | - | - | - | - | - | - | - |
| Adrenal, n, [%] | 12 (39) | 18 (37) | - | - | - | - | - | - | - |
| Ectopic, n, [%] | 7 (22) | 3 (6) | - | - | - | - | - | - | - |
| Serum cortisol (after 1 mg Dexamethasone), [μg/dl] | 14.3 (6.2, 21.2) | 1.0 (1.0, 1.3) | - | - | <0.001 | - | - | - | - |
| 24h urinary free cortisol [mcg/24h] | 194 (53, 385) | 41 (27, 78) | - | - | <0.001 | - | - | - | - |
| Glucocorticoid substitution n, [%] | 0 (0) | 35 (71 | - | - | <0.001 | - | - | - | - |
| *Comorbidities* |  |  |  |  |  | - | - | - | - |
| Hypertension n, [%] | 23 (74) | 24 (49) | - | 243 (82) | 0.002 | - | 0.695 | - | 0.001 |
| Diabetes mellitus n, [%] | 12 (39) | 6 (12) | - | 92 (31) | 0.001 | - | 0.015 | - | 0.007 |
| Hypothyreosis | 12 (39) | 23 (47) | - | - | 0.788 | - | - | - | - |
| Osteoporosis | 9 (29) | 9 (18) | - | - | 0.006 | - | - | - | - |
| *Medications* |  |  |  |  |  |  |  |  |  |
| Beta blocker, n [%] | 9 (29) | 10 (20) | - | 92 (31) | 0.079 | - | 0.617 | - | 0.344 |
| ACE or AT1 antagonist, n [%] | 16 (52) | 16 (33) | - | 138 (48) | 0.030 | - | 0.062 | - | 0.463 |
| Diuretics, [%] | 7 (23) | 6 (12) | - | 40 (15) | 0.075 | - | 0.051 | - | 0.987 |
| Aldosterone antagonist, [%] | 2 (7) | 1 (2) | - | - | 0.523 | - | - | - | - |
| *Echocardiography* |  |  |  |  |  |  |  |  |  |
| IVSd (mm) | 9 (1) | 9 (1) | 8 (1) | 10 (1) | 0.045 | <0.001 | 0.403 | 0.007 | 0.002 |
| LVEDd (mm) | 47 (5) | 45 (5) | 47 (5) | 50 (5) | 0.497 | 0.715 | 0.119 | 0.100 | 0.001 |
| LVPWd (mm) | 8 (1) | 8 (1) | 7 (1) | 9 (1) | 0.027 | <0.001 | 0.967 | 0.003 | 0.024 |
| LVEDV (mL) | 65 (17) | 81 (18) | 97 (25) | 106 (24) | 0.568 | <0.001 | <0.001 | <0.001 | <0.001 |
| E/e’ | 9 (3) | 9 (3) | 7 (2) | 9 (3) | 0.554 | <0.001 | 0.050 | <0.001 | 0.075 |
| Left atrial volume index (mL/m^2^) | 19 (15, 24) | 20 (15, 27) | 22 (18, 26) | 25 (20, 31) | 0.305 | 0.009 | 0.003 | 0.107 | 0.002 |
| LVEF [%] | 62 (4) | 61 (6) | 61 (4) | 60 (5) | 0.471 | 0.539 | 0.095 | 0.468 | 0.253 |
| GLS [-%] | 19.3 (2.5) | 20.4 (2,4) | 21.4 (2.4) | 19.1 (2.4) | 0.250 | <0.001 | 0.363 | <0.001 | 0.574 |

Data are n (%) or mean (SD) or median (interquartile) respectively. P-values adjusted for age and sex.

Abbreviations. BMI, body mass index; CS, Cushing Syndrome; DBP, diastolic blood pressure; E/e’, ratio between early mitral inflow velocity and mitral annular early diastolic velocity; GLS, global longitudinal strain; HbA1c, glycosylated hemoglobin; HS, healthy subjects; IVSd, interventricular septum diameter; LDL, low-density lipoprotein; LTR, long-term remission; LVEDd, left ventricular end-diastolic diameter; LVEDV, left ventricular end-diastolic volume; LVEF, left ventricular ejection fraction; LVPWd, left ventricular poster wall diameter; MS, metabolic syndrome; NT-proBNP, N-terminal pro-natriuretic peptide; SBP, systolic blood pressure.

**Supplementary Table 2:** Myocardial work in Cushing’s syndrome (either overt CS or in CS in long-term remission) and healthy subjects without or with metabolic syndrome.

|  | Overt CS n=31 (1) | CS-LTR n=49 (2) | STAAB  Healthy subjects n=439 (3) | STAAB  Metabolic syndrome n=293 (4) | 1 vs. 2 | 1 vs. 3 | 1 vs. 4 | 2 vs. 3 | 2 vs. 4 |
| --- | --- | --- | --- | --- | --- | --- | --- | --- | --- |
| GCW [mmHg%] | 2581 (455) | 2514 (378) | 2440 (334) | 2483 (423) | 0.426 | 0.001 | 0.505 | 0.012 | 0.208 |
| GWW [mmHg%] | 105 (74, 147) | 97 (69, 158) | 75 (54, 109) | 95 (65, 136) | 0.961 | 0.006 | 0.011 | <0.001 | 0.002 |
| GWI [mmHg%] | 2290 (387) | 2265 (378) | 2224 (310) | 2244 (400) | 0.207 | <0.001 | 0.057 | 0.001 | 0.741 |
| GWE [%] | 95 (2) | 95 (3) | 96 (2) | 95 (2) | 0.988 | 0.003 | 0.086 | <0.001 | 0.044 |

Data are n (%), mean (SD), or median (quartiles), respectively.

*p-values are obtained from analysis of covariance (ANCOVA) adjusted for age, sex, body mass index, and systolic blood pressure

Comparison versus overt CS: A p<0.05, B p<0.01, C p<0.001.
Comparison versus CS in LTR: a p<0.05, b p<0.01 c, p<0.001

CS, Cushing`s syndrome; GCW, global constructive work; GWE, global work efficiency; GWI, global work index; GWW, global wasted work; LTR, long-term remission.
